# Distribution of mammal functional diversity in the Neotropical realm: Influence of land-use and extinction risk

**DOI:** 10.1371/journal.pone.0175931

**Published:** 2017-04-25

**Authors:** José F. González-Maya, Enrique Martínez-Meyer, Rodrigo Medellín, Gerardo Ceballos

**Affiliations:** 1 Instituto de Ecología, Universidad Nacional Autónoma de México, México DF, Mexico; 2 Proyecto de Conservación de Aguas y Tierras, ProCAT Colombia, Carrera 13 # 96–82, Of. 205, Bogotá, Colombia; 3 Instituto de Biología, Universidad Nacional Autónoma de México, México DF, Mexico; Smithsonian Conservation Biology Institute, UNITED STATES

## Abstract

Functional diversity represents a measure of diversity that incorporates the role of species in an ecosystem, and therefore its dynamics and resilience. Assessing its drivers and spatial variation represents an important step forward in our understanding of functional ecosystem dynamics and it is also necessary to achieve a comprehensive conservation planning. In this paper, we assessed mammal functional diversity for the 218 ecoregions within the Neotropical realm. We evaluated the overall influence and spatial variation of species richness, ecoregion extent, intervention and species at risk on functional diversity. Using ordinary least squares and geographically weighted regression modeling approaches, we found that intervened areas and threatened and non-threatened species are the most influential overall drivers of functional diversity. However, we also detected that these variables do not operate equally across scales. Our local analyses indicated both that the variation explained and local coefficients vary spatially depending on the ecoregion and major habitat type. As estimates of functional diversity are based on current distribution of all mammals, negative influence of intervened areas and positive influence of non-threatened species may reflect a potential degradation of functional processes for some ecosystems. Most generally, the negative influence of intervention together with the influence of threatened species indicates that some areas are currently more susceptible to functional diversity loss. Our results help to pinpoint key areas requiring urgent conservation action to reduce natural land-cover loss and areas where threatened species play influential roles on ecosystem functioning.

## Introduction

Biological diversity has been historically measured via multiple approaches, ranging from basic counts of species richness to more sophisticated measures of evolutionary and functional diversity [1–4]. As more refined and complex measures arise, more aspects of community and ecosystem dynamics are assessed, allowing more precise inferences about ecosystems functioning, resilience and services [2, 5], and in turn, better conservation planning [6]. The Neotropics is one of the most diverse and complex regions in the world [7, 8], but also one of the most threatened due to anthropogenic factors [4, 9]. As human intervention increases in Neotropical ecoregions, more precise, effective and efficient conservation measures are needed, and at better resolution, in order to reduce biodiversity loss and thus preserve ecosystem services [10, 11]. Understanding underlying drivers of ecosystem function and diversity may allow designing better conservation actions [12, 13].

Disturbance has been previously identified as a determinant and change driver on multiple diversity measures, including functional diversity, and in general on ecosystem functioning for multiple taxa and ecosystems across the globe [13–16]. In fact, functional diversity has been proposed as a useful measure for comparing different ecological scenarios, such as land use change [14], reflecting its effect on ecosystem functioning. The effect of this disturbance, mostly in terms of habitat loss, varies spatially and taxonomically [13–16], and previous studies have determined that species diversity is a good proxy of ecosystem stability but varies according to the ecosystems´ disturbance history [13]. Since increasing disturbance can have a significant effect on ecosystem stability, understanding which regions have suffered, or are suffering, more significant functional loss is critical in order to prevent or stop such degradation. Furthermore, species loss occurs not only due to habitat loss but also to multiple factors operating over species and populations [17, 18]. Species at risk, namely those with higher likelihood of disappearing on the short term, also play important ecological roles and their disappearance would be reflected on the loss of functional complementarity and therefore on ecosystem function [19]. The combination of the effect of human disturbance and species at risk would then cover all potential likelihood drivers of functional loss, providing a better idea of which regions are likely on higher risk of suffering significant functional degradation [6, 19, 20].

Considering that almost no baseline information exist for most taxonomic groups in the Neotropics [21], and it was not until recently that complete groups were assessed in terms of their conservation status and distribution information [11, 22–24], assessing changes and variation, or original conditions, in terms of ecological processes and extinction occurring at large scales is a difficult task [21]. Some previous analyses assessed the magnitude of changes in terms of land-cover for the Neotropics [25, 26], but to our knowledge few studies have related these changes to ecosystem functionality or functional diversity as for other regions [3, 13–15]. Furthermore, so far, mammal diversity has been mostly studied from the macroecological perspective at global scales [7, 10, 17], with very few analyses assessing mammal diversity at regional/national scales [3, 4, 27–29].

Here we provide a regional-scale analysis of mammal taxonomic and functional diversity distribution as a basis for developing adequate conservation planning. Our specific goal was to assess the relationship between mammal functional diversity and human intervention/effects in the Neotropical ecoregions and its implications for conservation. We specifically address the following questions: i) how is species richness and mammal functional diversity distributed across the Neotropical realm and how is it distributed according to similar major habitat types?, ii) what is the relationship between land-cover, ecoregion area and species extinction risk on mammal functional diversity?, (iii) what is the spatial variation of these relationships?, and (iv) what are the critical ecoregions for conservation action based on mammal functional diversity?

## Materials and methods

We assessed mammal species richness and functional diversity for all 218 Neotropical ecoregions and 11 biomes (S1 Table) according to WWF ecological regionalization of the world [30], excepting St.Peter-St.Paul Rocks. We used the ecoregions approach based on numerous reasons: (1) ecoregions represent similar specific ecological continuums [30] that (2) have close relationship with taxonomic and functional compositions [3, 31, 32], thus having ecological significance, and (3) should better reflect the effect of species composition and intervention on functionality [13–15, 31, 33]; (4) patterns of disturbance and transformation occur similarly along similar biomes and ecoregions, most of the times with similar underlying drivers [27, 33]; (5) the median size (area) of all Neotropical ecoregions (Σ16,000 km^2^) does not significantly differ from most continental-scale ecological analyses resolution (1°x1°, Σ12,200 km^2^ in the tropics) [34]; (6) this resolution has been previously used for similar analyses [27, 35]; and, in terms of conservation planning, (7) allows better prioritization given the ecological significance of such regionalization, and not necessarily responding to political or arbitrary boundaries but ecologically-significant spatially defined units [32]. In general, our approach is based on the fact that Functional Diversity (FD) is a result of evolutionary, ecological and functional processes occurring over long periods of time; this reflects community and functional structure related with ecosystem type and with geographical/ecological significance [3]. We overlapped the maps of all ecoregions and mammal distributions, derived from current distribution polygons of all mammals obtained from the IUCN Red List [7], identifying all species present in each ecoregion. These maps were selected as they represent a unique source comprising systematic information for most mammals and corrected by region and expertise [7]. We estimated species richness as the total number of mammal species present on each ecoregion. To evaluate functional diversity, we first compiled life-traits for all mammals present in all ecoregions (i.e., 1593 spp.), including trophic guild (i.e., carnivore, omnivore, herbivore), habit mode (i.e., volant, arboreal, terrestrial, fossorial, and aquatic) and body size (i.e., body mass), derived from PanTHERIA [36] and other databases [3, 18]; these traits were selected since they adequately resume various aspects of space and resource use, thus defining important niche dimensions of each species [3, 37, 38]. Body size has been previously used in many ecological studies because its close relationship with nearly all aspects of a species´ ecology and even to diversity distribution patterns (e.g., environmental filtering) within ecosystems [39, 40]. This trait in relationship with trophic and habitat use characteristics defines resources use in terms of quantity, type and how to acquire them [37]. This combination and their interactions are a good proxy of species roles and complementarity [41] and are likely highly related with the biodiversity and ecosystem function relationship [38]. Furthermore, these traits are available for most species [18] and have been previously informative for exploring risk and functional diversity in mammals [3, 4, 37, 38].

### Mammal diversity measures

**Fig 1:**
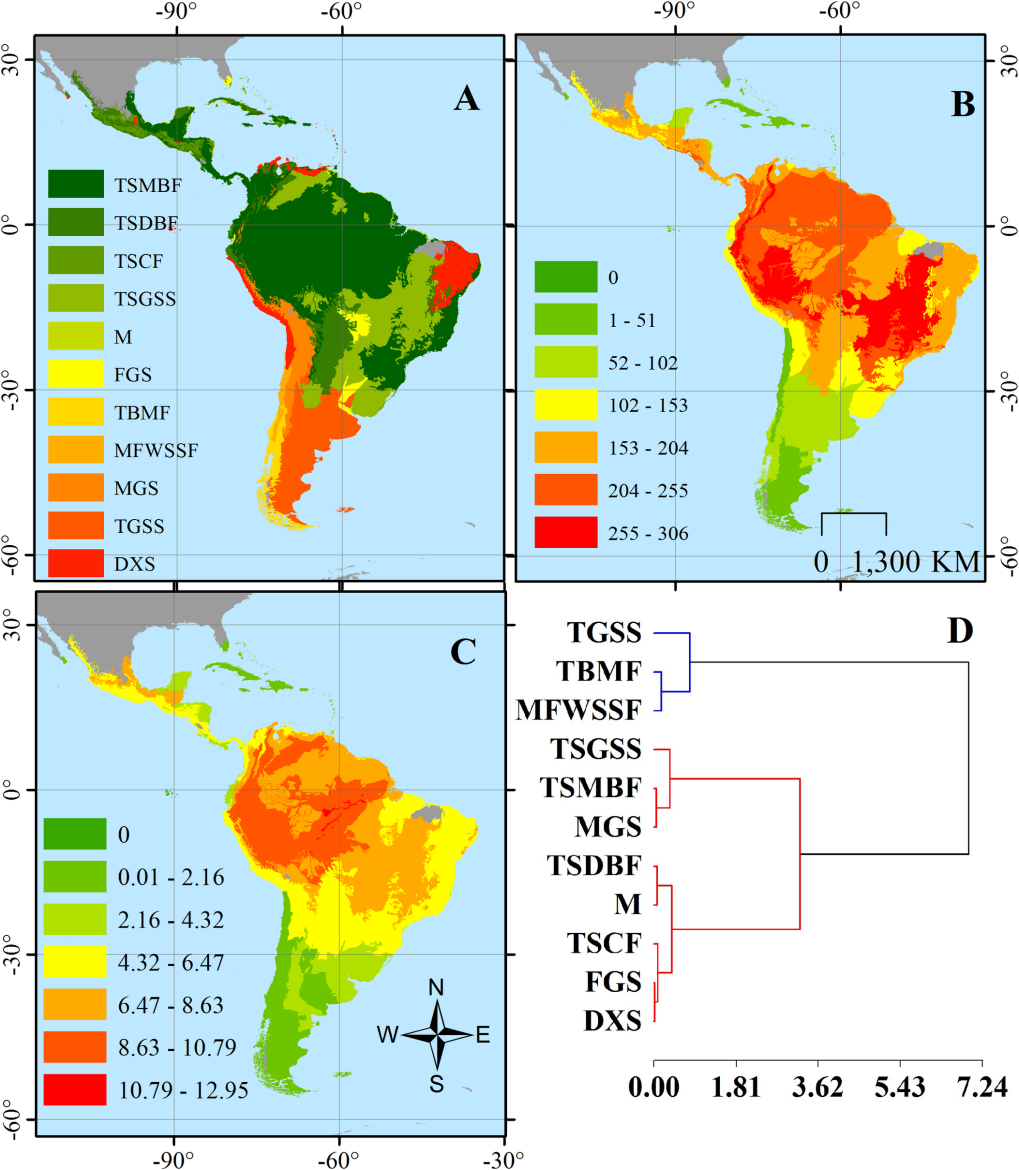
**Ecoregions of the Neotropical realm indicating (A) major habitat types (biomes) distribution, (B) species richness and (C) functional diversity and (D) Cluster analyses for ecoregions classified according to major habitat type (i.e. biome) based on species richness and functional diversity values.** Euclidian squared distance and average linking was the best linking method according to cophenetic correlation (0.668).

Once a species list for each ecoregion was obtained, we estimated species richness and a FD index based on these traits. The FD index was calculated based on Petchey and Gaston (2002) index, which is defined as the sum of the dendrogram branch distances necessary to connect all species in the functional space [37, 42]. We used this metric because it has been previously shown to perform well with multiple traits (i.e., nominal and quantitative [3, 43]), it interacts with species richness but performs particularly well for species rich communities–such as those in the Neotropics, and it does not depend on abundance data [3, 44]. We estimated a distance matrix and functional dendrogram based on the Gower distance (i.e., Unweighted pair group with arithmetic averages) and summed the branches necessary to connect all the species present in the ecoregion. The FD index considers that complementarity is high when the index is comparatively high, thereby indicating species are distant in trait-space, and low complementarity occurs when the index is comparatively low, indicating species are more similar [37, 45]. We assessed variation and similarities of species richness and FD across ecoregions and according to corresponding biomes. To do so, we used ecoregion classification according to major habitat types [30], classifying each in eleven biomes: Tropical and subtropical (T&S) moist broadleaf forests (TSMBF), T&S dry broadleaf forests (TSDBF), T&S coniferous forests (TSCF), T&S grasslands, savannas, and shrublands (TSGSS), Mangroves (M), Flooded grasslands and savannas (FGS), Temperate broadleaf and mixed forests (TBMF), Mediterranean forests, woodlands, and scrub-sclerophyll forests (MFWSSF), Montane grasslands and shrublands (MGS), Temperate grasslands, savannas, and shrublands (TGSS), and Deserts and xeric shrublands (DXS; Fig 1A). Then, we assessed normality using a Shapiro-Wilk test, and subsequently performed a Kruskal-Wallis non-parametric test to determine differences on species richness and functional diversity values, and post-hoc tests for identifying the biomes with higher FD and richness values [46]. In order to evaluate similarities between biomes according to species richness and FD, we performed a cluster analysis with square Euclidean distances and different linking methods, selecting those with the highest cophenetic correlation [47].

### Drivers of mammal functional diversity

In order to assess the influence of macroecological and anthropogenic variables and species at risk on FD, we used Ordinary Least Squares modeling approach (OLS; [3]). OLS is a generalized linear modelling technique homologous to linear regressions, accounting for minimizing the sum of the squares of the differences between the predicted linear values and those observed on the dataset, thus indicating a causal relationship [48, 49]. We considered four main variables accounting for gradients and ecological, intervention and risk dimensions identified by previous studies [1, 3, 13, 37, 50]. The variables included were: 1) mammal species richness, defined as the total number of species present in each ecoregion since it is expected this is the most important driver of FD [45]; 2) the area of the ecoregion, defined as its geographic extent [30], considering it has been previously identified as a driver of FD and trait richness [51]; 3) intervention, defined as the area of natural and artificial land-covers derived from the GlobCover database [52], based on previous analyses indicating the influence of disturbance on FD [3, 13–15], and; 4) the number of species classified as threatened and non-threatened present in the ecoregion, derived from the IUCN Red List of Threatened Species [53], classifying those species assessed as Least Concern and Near Threatened as non-threatened and those assessed as Data Deficient, Vulnerable, Endangered and Critically Endangered as threatened, and based on the assumption that species at risk will likely disappear sooner, therefore more rapidly affecting FD [50]. Even when other human intervention variables potentially affect FD, generally assessing their influence on diversity measures at multiple scales is challenging due to the generalized absence of spatial information at appropriate resolutions [11, 54]. Land-use change, in the form of habitat loss and fragmentation, is considered the most influencing driver of species extinction risk, particularly in mammals [7]; therefore, even when we did not include other variables explicitly, most of them are implicitly considered as the main factors driving a species towards extinction. Our selection of potential influencing variables was based on allowing an accurate assessment of the effect of known and measurable variables over FD, thus allowing better definition of priority areas and implementation of actions.

We performed models with all possible variable combinations and no interaction terms (i.e., 68 combinations), and selected those with the lower Akaike Information Criterion value (AIC) corrected for small samples and AIC weights (AICw; [55]). We assessed model performance based on both the AICc and the adjusted R^2^ (adjR^2^), and selected the best fitting model with significant predictive variables. In order to assess correlation of influencing variables, we estimated Variance Inflation Factors (VIF) for each variable and assessed large VIFs (>7) according to the model as potentially correlated with other variables [56]. In cases where a variable showed high VIF values in a large number of models, we estimated Spearman correlation coefficients to determine which variables were highly correlated, thus similarly informing the models. For assessing the significance of the variables, we estimated Koenke studentized Breusch-Pagan statistic (K(BP)) and its probability, in order to assess the reliability of standard errors when heteroskedaticity is present; in case the K(BP) was significant, we used the Robust Probability instead of the raw probability estimation. Jarque-Bera statistic and Moran´s I were used for testing for residuals normality and clustering, respectively; in case residuals were clustered, we generated a spatial weights matrix and included the weights in the model. The K(BP) statistic also assess the non-stationarity of the model, meaning that explanatory variables do not behave the same everywhere (i.e., overall OLS model), therefore indicating that their influence does not operate homogeneously across geographic space [57].

### Spatial variation of variables´ influence on functional diversity

After identifying non-stationarity, we performed a Geographic Weighted Regression (GWR) with the selected variables, allowing us to assess if local models performed better than the overall OLS model by comparing Pseudo R^2^ values [58]. Also, we assessed the local explanatory magnitudes (adjR^2^) and coefficients for each variable over each ecoregion, assessing where stronger influence relationships occur for all variables (adjR^2^) and where certain variables are more important or negatively/positively influence FD (local coefficients; [3, 59, 60]). GWR evaluates the influence of variables for each ecoregion by generating individual models using a defined number of adjacent ecoregions (i.e., neighbors); for selecting the number of neighbors we used an adaptive corrected AIC method with cross-validation, considering that ecoregions are distributed continuously across the Neotropical realm, and aiming to minimize AIC values [59]. We used the condition number as a diagnostic of local multicollinearity, indicating a locally unstable model; as a rule-of-thumb, condition numbers larger than 30 are likely unreliable. To test for the generalized FD explained by selected variables derived from local relationships according to biomes, we included all biomes as prediction localities when defining the model in order to compute coefficients and weighted R^2^ for each biome. Also, we used the generated condition numbers as an indication of unstable models and multicolinearity.

### Ecoregions of high conservation priority

To select the ecoregions of high conservation priority we identified those ecoregions that are more influenced by threatened species, according to estimated coefficients, and divided the coefficient values in three classes (i.e. low, medium and high priority). We mapped these ecoregions and spatially identified those as priority for conservation action.

All geographic and statistical analyses were performed in ArcGIS 10.2 [61] and R environment [62].

## Results

### Mammal species richness and functional diversity distribution

Mammal FD and species richness across all Neotropical ecoregions showed a gradient distribution from the lowest values in the Southern Cone towards higher values in the Central and Northern regions of South America, Mesoamerica and decreasing towards Central Mexico and the Caribbean (Fig 1B). Both measures showed a non-parametric distribution (Shapiro-Wilks; W = 0.96, p<0.0001 and W = 0.95, p<0.0001, respectively) with a dominance of lower values in most ecoregions. There was significant variation in FD (Kruskal-Wallis; H = 44.71, p<0.0001) and species richness (H = 48.80, p<0.0001) among major habitat types (i.e., biomes). Four biomes (T&S Dry Broadleaf Forests, Grasslands, Shrublands and Savannas and Moist Broadleaf Forests, and Montane Grasslands and Shrublands) had the highest mean (±SD) species richness (116±73, 148±89, 160±72 and 169±61, respectively) and mean (±SD) FD values (4.14±2.44, 5.09±3.11, 5.77±2.855, 6.02±2.17, respectively). Both measures tended to increase with decreasing latitude (Fig 1B), with greater FD values towards the Amazon basin (Fig 1C). Two main clusters with one subdivision were identified among biomes according to measures (Squared Euclidean Distance, average linkage; Cophenetic correlation = 0.668). One group included Temperate Grasslands, Savannas, and Shrublands, Temperate Broadleaf and Mixed Forests and Mediterranean Forests, Woodlands, and Scrub or Sclerophyll Forests, all temperate biomes; the second cluster was divided in two groups, one consisting on T&S Grasslands, Savannas, and Shrublands, Moist Broadleaf Forests, and Montane Grasslands and Shrublands; and a second group including T&S Dry Broadleaf Forests, Coniferous Forests, Mangrove, Flooded Grasslands and Savannas, and Deserts and Xeric Shrublands (Fig 1D). Variation in both measures responded significantly to the major habitat type (i.e., biomes).

### Mammal functional diversity influencing factors

Mammal FD showed a significant relationship with three variables across the entire Neotropical realm, while ecoregion area did not have a significant effect on this measure. The best selected model indicated a positive relationship between both threatened and non-threatened species, more species increases FD, and a negative relationship with the intervened area of the ecoregion, indicating that when the area of degraded land-covers increases, FD tends to decrease (Table 1). The first variable excluded from the model, despite the fact that it was used in all possible combinations, was species richness, as expected from previous studies and the FD index used, since it showed a significant correlation with other determinant variables (i.e. threatened and non-threatened species), potentially clouding the effect of the other determinants (Spearman correlation = 0.92, p<0.001). Our model indicated a significant FD variation explained by the predictor variables (R^2^ = 0.872), and residuals tested for normality (JB = 84.6, p = 0.08) and non-clustering (Moran´s I = 0.03, Z-score = 0.36), thus not needing the use of spatial weights for correcting the model. The model also showed non-stationarity, indicating that the relationships found do not remain constant across space and scale (K(BP) = 19.69, p<0.001), therefore, robust probabilities were used for all variables (Table 1).

**Table 1:** Best performing selected model including the selected determinant variables influencing mammal functional diversity in the ecoregions of the Neotropical realm. AIC: Akaike Information Criterion, adjR^2^: adjusted R^2^, AICw: AIC weights and VIF: Variance Inflation Factor.

Model	Parameters	Coef.	VIF	Robust p	AIC	adjR^2^
1	Intercept	0.244	-	<0.001	606.15	0.872
	Intervened	-2.46E-07	1.17	<0.001		
	Threatened	0.022	3.80	0.026		
	Non-threatened	0.029	3.56	<0.001		

### Spatial variation of the relationship between variables and functional diversity

**Fig 2:**
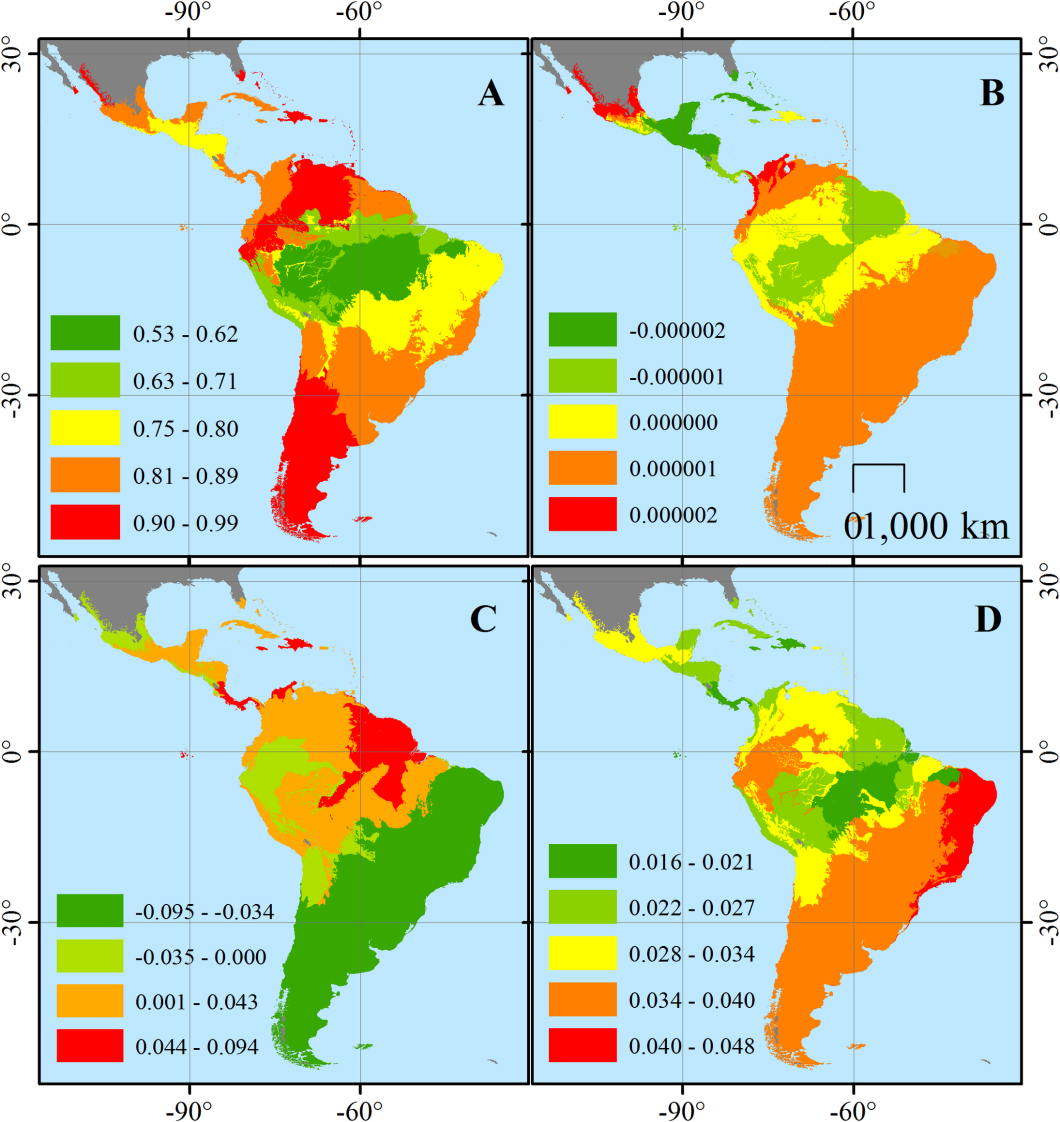
**Local values of (A) variation explained by selected variables–R**^**2**^ **and coefficients for (B) intervened land use, (C) threatened and (D) non-threatened species influencing mammal functional diversity for each ecoregion of the Neotropical realm based on a geographically weighted regression.** Higher R^2^ values indicate higher functional diversity variation explained by the selected variables; higher coefficient value indicates higher positive influence of each variable while negative values indicate negative influence on functional diversity values.

The geographically weighted regression (GWR) indicated a differential relationship according to the spatial location of each ecoregion. The weighted model (Pseudo R^2^ = 0.98) performed better than the overall OLS model (Pseudo R^2^ = 0.89), and the variation explained by all influencing variables also increased significantly (adjR^2^ = 0.9248). The variability explained for each ecoregion “neighborhood” varied significantly across the Neotropical realm, with varying R^2^ values between 0.53 and 0.99 (mean±SD = 0.84±0.09); Northern South America, and the South Cone showed the strongest relationship between FD and species, threatened and non-threatened, and intervention, while the lowest values were located in the southern Amazon basin (Fig 2A).

Condition numbers indicated no multicollinearity among the local models for each ecoregion (Mean±SD = 12.28±4.56). In terms of the influence of each variable across each ecoregion, we found that intervention negatively influenced FD more notably in the Amazon Basin and Northern Mesoamerica (Fig 2B) with overall low intervention coefficients (mean coefficient±SD = -4.86E-07±9.64E-07). Threatened species most significantly influenced positively in the Guyanas and influenced negatively in South America’s Atlantic coast (mean coefficient±SD = 0.006±0.042; Fig 2C). Finally, non-threatened species had higher influence in the Caatinga and Northern Brazil and lowest influence for the Southern Amazon basin (mean coefficient±SD = 0.03±0.007; Fig 2D). Overall explained variation weighted and projected by biomes was high (R^2^ mean±SD = 0.93±0.03), where the highest influence was determined for Temperate grasslands, savannas, and shrublands and the lowest for Flooded grasslands and savannas; no multicollinearity was found for local biome models (Table 2). T & S Broadleaf Moist Forests, Dry Broadleaf Forests, and Montane Grasslands and Mangroves were the biomes more significantly affected both by human intervention and species risk, while the ecoregions located in Northern South America, Mexico and Southern Argentina and Chile were the ones suffering the strongest influence of only intervention on FD. Condition numbers of weighted models for each biome showed stability and no effects of multicolinearity (Mean±SD = 8.18±2.56).

**Table 2:** Local explanatory magnitude (R^2^) and intervened, threatened and non-threatened species coefficients influence over mammal functional diversity weighted and projected for each major habitat types (i.e., biome) in the Neotropical realm. TSBMF: Tropical and subtropical moist broadleaf forests, TSDBF: Tropical and subtropical dry broadleaf forests, TSCF: Tropical and subtropical coniferous forests, M: Mangroves, TBMF: Temperate broadleaf and mixed forests, TSGSS: Tropical and subtropical grasslands, savannas, and shrublands, TGSS: Temperate grasslands, savannas, and shrublands, FGS: Flooded grasslands and savannas, MGS: Montane grasslands and shrublands, MFWSSF: Mediterranean forests, woodlands, and scrub or sclerophyll forests and DXS: Deserts and xeric shrublands.

Biome	Condition numbers	Local R^2^	Coefficients		
			Intervened	Threatened	Non-threatened
TSBMF	10.32	0.91	-1.4E-08	-0.04	0.04
TSDBF	10.49	0.93	-1.2E-08	-0.02	0.04
TSCF	6.26	0.94	1.9E-06	0.01	0.03
TBMF	4.82	0.99	-4.8E-07	0.04	0.03
TSGSS	10.95	0.89	5.1E-08	-0.11	0.05
TGSS	5.24	0.99	-4.5E-07	0.04	0.03
FGS	10.28	0.89	2.2E-08	-0.09	0.05
MGS	10.01	0.94	2.0E-08	-0.04	0.04
MFWSSF	9.76	0.90	4.9E-08	-0.09	0.05
DXS	9.57	0.92	5.0E-08	-0.07	0.05
M	10.22	0.93	-2.0E-09	-0.02	0.04

### Priority ecoregions for conservation action

**Fig 3:**
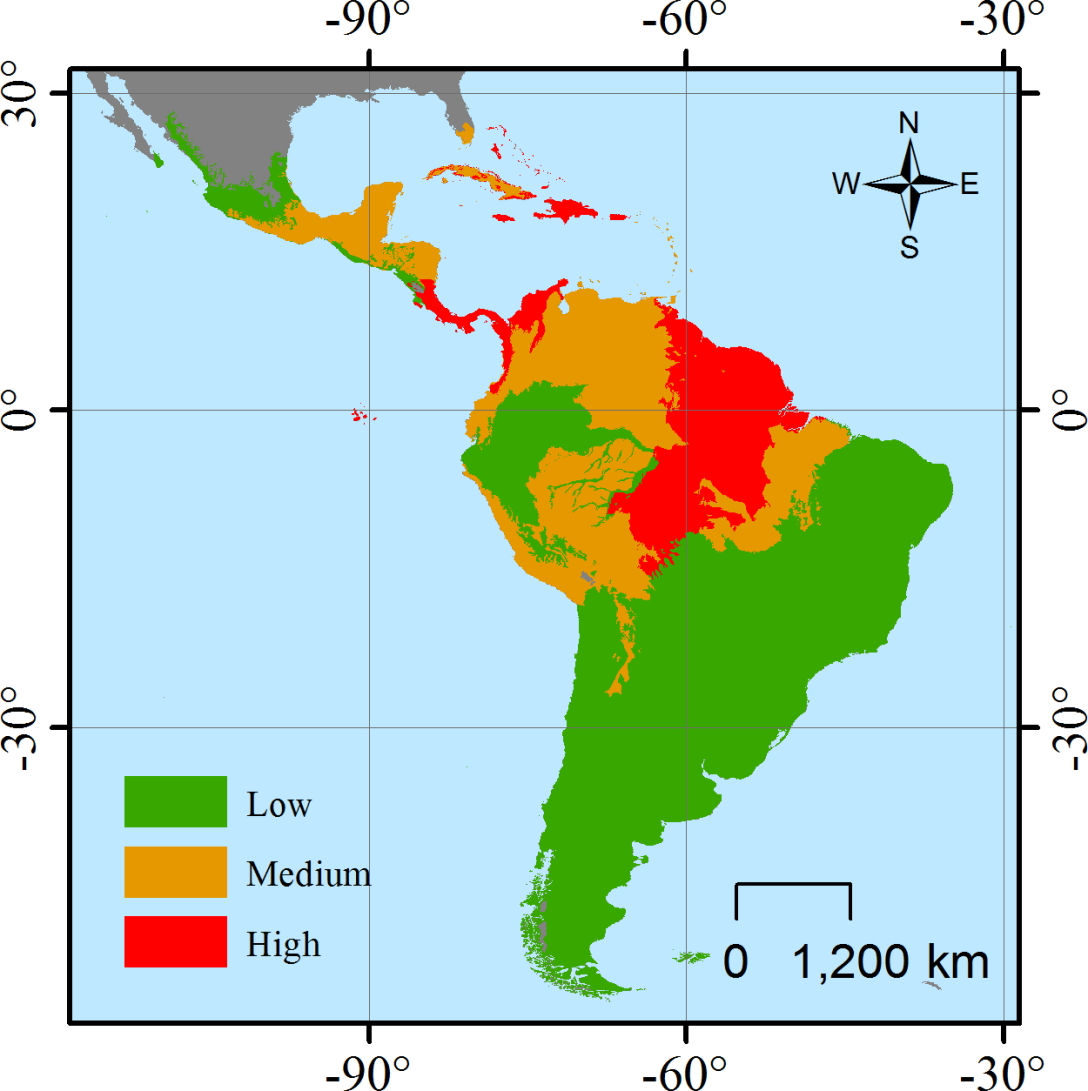
Priority ecoregions for mammal functional diversity change according to priority ecoregions were mammal functional diversity is more significantly influenced by threatened species therefore more susceptible to species loss for the Neotropical realm.

We identified 49 high priority ecoregions where species at risk significantly influences FD (S1 Table); most of the ecoregions are located in the Caribbean, the continuum between southern Nicaragua throughout Costa Rica and Panama into the Caribbean, Choco and Magdalena valley in Colombia, and the Guyana shield in South America (Fig 3). The biomes with higher estimated influence of threatened species were the T&S Coniferous, Temperate Broadleaf Forests and Tropical Grasslands (Table 2) while intervention is more significantly reducing functional diversity for T&S Moist and Dry Forests, Temperate Forests, Tropical Grasslands and Mangroves (Table 2).

## Discussion

Mammal functional diversity (FD) across the Neotropical realm showed considerable variation across most ecoregions, but was very similar across major habitat types (Fig 1D); also, it was significantly influenced both by human impact and species at risk. Differences in FD among major habitat types probably reflect natural differences due to habitat and environmental constraints, which affects both ecosystems and animal assemblages through environmental filtering [63, 64]. Furthermore, similarities among habitats of the same type (e.g., T&S forests) highlight how this filtering is potentially defining ecosystem structure in terms of functional types or species roles [4].

Our results highlight the importance of anthropogenic intervention, both at the ecosystem and species levels (i.e., disturbance and species at risk), regarding ecosystem resilience and dynamics [65, 66]. Also, our results indicated geographic variation of the influence of the explanatory variables, indicating the large variation along small space gradients characteristic of the Neotropics [3, 67], but also, in terms of anthropogenic factors: how certain ecoregions are under more severe threat of losing functionality than others. The results of the model are robust (i.e., high variation explained) in terms of explaining the variation of FD as defined by species richness (i.e., species threatened and non-threatened) and current human intervention. Overall model variation explained by selected variables, and geographically weighted maximum variation explained for certain ecoregions, indicates that some regions are heavily affected by human intervention; however, for ecoregions with low levels of intervention only ~50% of this variation was explained.

Previous studies of global and regional variation on FD in mammals and other groups have shown similar patterns than those obtained here [3, 4, 37, 68, 69], and most studies have identified anthropogenic intervention–in terms of land-cover transformation, as a major driver for FD loss [14, 15, 38, 70]. However, very few studies have assessed the influence of species at risk and the potential functional loss due to species extinctions [38, 50, 71]. Our results support the premise that human intervention and species at risk can significantly alter ecosystem function [38, 50], but also, highlight how threatened species, that according to IUCN standards are mostly those restricted, vulnerable or rare, contribute significantly (i.e., complementarity) to unique ecosystem functions [72, 73]. Furthermore, the impact of ecosystem functionality loss has deeper implications in terms of ecosystem resilience and vulnerability [6, 12, 74]; species loss not only means a reduction in the number of species in an ecosystem, but also serious impacts at the community and ecosystem levels [17].

Species richness analyses have previously shown high correlation with FD [75], especially when using our metric [3, 45], therefore it was expected these variables were highly related [3, 4]. Nevertheless, given the complete assessment of the group [7], species richness influence could be divided between threatened and non-threatened species, allowing to isolate and identify the potential negative impact of species loss. The strong influence of intervention and species at risk on FD in certain ecoregions and biomes helps to identify specifically where priorities should be focused in order to reduce species and ecosystem functionality loss [6, 15, 17, 38, 50]. Biomes and ecoregions at higher risk, as identified by our model, demand prior attention since impacts of functional loss affect beyond biological diversity, it reduces ecosystem resilience and services provision [6, 12, 74]. Our identification of priority ecoregions by influence of species at risk, indicate how certain areas require more urgent conservation actions at species, populations and ecosystem levels, so FD, and thus ecosystem functioning, can be maintained. Most of the ecoregions encompassing most of the Neotropical human population are those requiring reduction of deforestation and intervention in general. Ecoregions in the likely most diverse areas of the Neotropics are those requiring species-level protection since threatened species considerably influence FD and hence ecosystem function.

The coarse resolution distribution maps for certain species, and the intrinsic limitations from the index, functional grouping and diversity estimations being relatively new for mammals, and animals in general [76], may give rise to some constraints of our approach. However, most of the future discussion relies on which life and ecological traits are more relevant or informative for functional roles of species in ecosystems. In addition, our approach still highlights that even when few traits are used, human intervention is a severe threat to FD, and it is likely that future analyses at finer resolutions will show the same trend, since it is expected that lower redundancy will be observed among species [71, 77]. Because we analyzed current mammal distribution ranges, positive influence of intervention can be related to already-degraded assemblages or already-affected FD, suggesting that common species play more significant key roles in ecosystems than rare species; this trend coincides in those ecoregions where non-threatened species have the highest influence.

Conservation applicability of our results are both spatially-defined, by focusing on the priority ecoregions, and conservation action-defined, by focusing efforts on the highest threat to functional stability. T&S Coniferous, Temperate Broadleaf Forests and Tropical Grasslands are priority for improving species management and reducing the risk of those already considered under threat. T&S Moist and Dry Forests, Temperate Forests, Tropical Grasslands and Mangroves ecoregions are those identified as priority for urgently reducing or mitigating habitat loss derived from land-use change. Previous efforts have already prioritized areas based on the number of species at risk or simply using measures of species richness [7, 34, 78, 79] while others have identified those areas with the highest rates of habitat loss [80, 81], however, our exploration of the link between human intervention and species at risk with ecosystem stability and function provides more precise tools for supporting where and how to focus conservation actions [14].

## Conclusions

This is among the first studies assessing the influence of human impacts over mammal FD, especially for one of the most diverse areas globally. Further explorations into mammal FD at finer and more detailed resolutions and more precise definitions of human intervention would likely provide confirmation of our results and will increase the precision of the trends found in our results [3, 4]. Our definition of FD, as linked to the concept of functional richness, is the functional trait space occupied by species within a community [75], influencing productivity and in turn functionality and resilience at ecosystem and community scale. Therefore, despite our coarse assessment of FD, influence of driving variables will definitely have significant conservation implications. Definition of conservation priorities at regional scales have proved an efficient tool to effectively tackle biodiversity loss, and region-wide assessments and actions may result in threat reduction and better conservation planning [27, 82, 83]. Our results help refine conservation planning analyses by providing new insights and tools on different biodiversity measures, especially by linking both species risk and human intervention with ecosystem vulnerability and resilience [6]. Previous priority-selection schemes have identified priority areas based on species richness, threat or singularities [9]; our approach does not undermine these initiatives by proposing new priorities, instead call for attention to those regions suffering functionality loss due to intervention and species loss. This will likely improve conservation actions not only for selecting conservation areas but as a tool for conservation planning and decision making by selecting adequate cost-benefit policies [84]. Also, this study can help enhance our understanding on how natural and induced spatial variability can be incorporated in conservation planning to improve our conservation practices aiming to reduce biodiversity loss, which is the current paramount environmental problem worldwide.

## Supporting information

S1 Table Neotropical ecoregions classified by biome (i.e. major habitat type), including ecoregion size (area), species richness (SR) and functional diversity index values (FD), and classified according to priority based on the influence of threatened species over mammal functional diversity. (DOCX)
