# Supplementary material for: Distribution of mammal functional diversity in the Neotropical realm: Influence of land-use and extinction risk
Source: PLoS One. 2017 Apr 25;12(4):e0175931. doi: 10.1371/journal.pone.0175931 (PMC5404856; doi:10.1371/journal.pone.0175931)
Supplement: S1 Table — (DOCX) [file pone.0175931.s001.docx]

**Supporting information**

**Table S1.** Neotropical ecoregions classified by biome (i.e. major habitat type), including ecoregion size (Area), Species Richness (SR) and Functional Diversity Index Values (FD), and classified according to priority based on the influence of threatened species over mammal functional diversity.

| **Biome** | **Ecoregion** | **Area**  **(km^2^)** | **SR** | **FD** | **Influence of** | | |  |
| --- | --- | --- | --- | --- | --- | --- | --- | --- |
|  |  |  |  |  | **Threatened species** | | |  |
|  |  |  |  |  | **Low** | **Medium** | **High** | |
| T&S moist broadleaf forests | Alta Paraná Atlantic forests | 482703.58 | 214 | 6.25 | X |  |  | |
|  | Araucaria moist forests | 215679.83 | 152 | 4.60 | X |  |  | |
|  | Atlantic Coast restingas | 7850.76 | 182 | 5.52 | X |  |  | |
|  | Bahia coastal forests | 109310.87 | 156 | 4.49 | X |  |  | |
|  | Bahia interior forests | 229253.46 | 185 | 5.40 | X |  |  | |
|  | Bolivian Yungas | 90234.02 | 237 | 7.17 |  | X |  | |
|  | Caatinga Enclaves moist forests | 4776.59 | 115 | 5.64 | X |  |  | |
|  | Caqueta moist forests | 183372.19 | 207 | 7.72 | X |  |  | |
|  | Catatumbo moist forests | 22741.26 | 185 | 6.68 |  | X |  | |
|  | Cauca Valley montane forests | 31917.55 | 207 | 7.91 |  | X |  | |
|  | Cayos Miskitos-San Andrés & Providencia moist forests | 94.84 | 1 | 0.00 |  |  | X | |
|  | Central American Atlantic moist forests | 89143.43 | 173 | 4.29 |  | X |  | |
|  | Central American montane forests | 13251.86 | 173 | 4.29 |  | X |  | |
|  | Chiapas montane forests | 5759.20 | 148 | 4.57 |  | X |  | |
|  | Chimalapas montane forests | 2076.76 | 124 | 4.87 |  | X |  | |
|  | Chocó-Darién moist forests | 73310.18 | 215 | 4.98 |  |  | X | |
|  | Cocos Island moist forests | 24.78 | 1 | 0.00 |  |  | X | |
|  | Cordillera La Costa montane forests | 14282.61 | 171 | 6.14 |  | X |  | |
|  | Cordillera Oriental montane forests | 67581.90 | 257 | 9.65 |  | X |  | |
|  | Costa Rican seasonal moist forests | 10654.23 | 171 | 4.01 |  |  | X | |
|  | Cuban moist forests | 21340.29 | 27 | 1.19 |  |  | X | |
|  | Eastern Cordillera real montane forests | 102044.25 | 269 | 9.75 | X |  |  | |
|  | Eastern Panamanian montane forests | 3031.44 | 172 | 3.97 |  |  | X | |
|  | Fernando de Noronha-Atol das Rocas moist forests | 18.86 | 0 | 0.00 | X |  |  | |
|  | Guayanan Highlands moist forests | 336105.89 | 220 | 8.31 |  | X |  | |
|  | Guianan Freshwater swamp forests | 7690.97 | 164 | 5.88 |  |  | X | |
|  | Guianan moist forests | 510640.64 | 208 | 7.67 |  |  | X | |
|  | Gurupa varzeá | 9881.49 | 164 | 8.28 |  |  | X | |
|  | Hispaniolan moist forests | 45815.77 | 16 | 0.64 |  |  | X | |
|  | Iquitos varzeá | 114543.30 | 238 | 9.36 | X |  |  | |
|  | Isthmian-Atlantic moist forests | 58674.61 | 198 | 4.69 |  |  | X | |
|  | Isthmian-Pacific moist forests | 29178.43 | 171 | 3.95 |  |  | X | |
|  | Jamaican moist forests | 8270.00 | 18 | 0.90 |  |  | X | |
|  | Japurá-Solimoes-Negro moist forests | 268464.04 | 221 | 9.09 |  | X |  | |
|  | Juruá-Purus moist forests | 241509.49 | 186 | 7.33 |  | X |  | |
|  | Leeward Islands moist forests | 986.52 | 14 | 0.79 |  | X |  | |
|  | Madeira-Tapajós moist forests | 716732.52 | 237 | 9.84 |  |  | X | |
|  | Magdalena Valley montane forests | 104605.94 | 238 | 9.05 |  | X |  | |
|  | Magdalena-Urabá moist forests | 76445.37 | 186 | 5.50 |  |  | X | |
|  | Marajó Varzeá forests | 88311.08 | 176 | 6.92 |  |  | X | |
|  | Maranhao Babaçu forests | 11518.00 | 170 | 5.48 |  | X |  | |
|  | Mato Grosso seasonal forests | 412336.15 | 179 | 7.45 |  | X |  | |
|  | Monte Alegre varzeá | 66510.16 | 230 | 12.95 |  |  | X | |
|  | Napo moist forests | 250612.35 | 249 | 8.95 | X |  |  | |
|  | Negro-Branco moist forests | 211912.74 | 206 | 7.50 |  | X |  | |
|  | Northeastern Brazil restingas | 10011.67 | 110 | 5.21 | X |  |  | |
|  | Northwestern Andean montane forests | 80810.12 | 243 | 7.22 |  | X |  | |
|  | Oaxacan montane forests | 7577.37 | 171 | 5.48 |  | X |  | |
|  | Orinoco Delta swamp forests | 28029.91 | 190 | 6.91 |  |  | X | |
|  | Pantanos de Centla | 17152.99 | 113 | 3.84 |  | X |  | |
|  | Pernambuco coastal forests | 17502.92 | 124 | 6.32 | X |  |  | |
|  | Pernambuco interior forests | 22598.69 | 130 | 6.59 | X |  |  | |
|  | Peruvian Yungas | 185974.05 | 271 | 10.01 | X |  |  | |
|  | Petén-Veracruz moist forests | 148604.73 | 203 | 6.65 |  | X |  | |
|  | Puerto Rican moist forests | 7504.54 | 11 | 0.64 |  |  | X | |
|  | Purus varzeá | 176775.54 | 237 | 9.52 | X |  |  | |
|  | Purus-Madeira moist forests | 173273.74 | 189 | 10.60 |  |  | X | |
|  | Rio Negro campinarana | 80502.33 | 185 | 7.13 |  | X |  | |
|  | Santa Marta montane forests | 4765.83 | 154 | 5.68 |  |  | X | |
|  | Serra do Mar coastal forests | 104613.50 | 177 | 5.03 | X |  |  | |
|  | Sierra Madre de Chiapas moist forest | 11218.27 | 138 | 5.63 | X |  |  | |
|  | Sierra de los Tuxtlas | 3890.32 | 102 | 3.12 |  | X |  | |
|  | Solimoes-Japurá moist forest | 166914.33 | 204 | 8.08 | X |  |  | |
|  | South Florida rocklands | 2070.97 | 35 | 1.39 |  | X |  | |
|  | Southern Andean Yungas | 60979.12 | 166 | 5.25 | X |  |  | |
|  | Southwest Amazon moist forests | 746704.18 | 306 | 9.07 |  | X |  | |
|  | Talamancan montane forests | 16275.58 | 186 | 4.38 |  |  | X | |
|  | Tapajós-Xingu moist forests | 335123.88 | 173 | 6.96 |  |  | X | |
|  | Tepuis | 48631.78 | 214 | 7.99 |  | X |  | |
|  | Tocantins/Pindare moist forests | 192778.42 | 152 | 5.55 |  | X |  | |
|  | Trindade-Martin Vaz Islands tropical forests | 10.90 | 0 | 0.00 | X |  |  | |
|  | Trinidad and Tobago moist forests | 4722.31 | 79 | 2.56 |  | X |  | |
|  | Uatuma-Trombetas moist forests | 471007.84 | 220 | 8.78 |  |  | X | |
|  | Ucayali moist forests | 114450.89 | 226 | 8.67 | X |  |  | |
|  | Venezuelan Andes montane forests | 29269.37 | 190 | 7.08 |  | X |  | |
|  | Veracruz moist forests | 68949.79 | 156 | 6.85 | X |  |  | |
|  | Veracruz montane forests | 4942.65 | 120 | 5.06 | X |  |  | |
|  | Western Ecuador moist forests | 33955.78 | 148 | 3.93 |  | X |  | |
|  | Windward Islands moist forests | 2011.91 | 25 | 0.65 |  | X |  | |
|  | Xingu-Tocantins-Araguaia moist forests | 265091.86 | 163 | 6.23 |  | X |  | |
|  | Yucatán moist forests | 69485.48 | 98 | 3.46 |  | X |  | |
| T&S dry broadleaf forests | Apure-Villavicencio dry forests | 68251.93 | 237 | 8.62 |  | X |  | |
|  | Atlantic dry forests | 114667.38 | 150 | 5.90 | X |  |  | |
|  | Bahamian dry forests | 4784.77 | 10 | 0.56 |  |  | X | |
|  | Bajío dry forests | 37282.25 | 131 | 5.71 | X |  |  | |
|  | Balsas dry forests | 62249.99 | 167 | 5.42 | X |  |  | |
|  | Bolivian montane dry forests | 80088.00 | 218 | 6.60 | X |  |  | |
|  | Cauca Valley dry forests | 7313.33 | 182 | 6.56 |  | X |  | |
|  | Cayman Islands dry forests | 132.86 | 4 | 0.77 |  | X |  | |
|  | Central American dry forests | 67777.54 | 212 | 5.08 | X |  |  | |
|  | Chaco | 608130.78 | 191 | 5.71 | X |  |  | |
|  | Chiapas Depression dry forests | 13974.51 | 153 | 4.73 |  | X |  | |
|  | Chiquitano dry forests | 229780.77 | 194 | 6.00 | X |  |  | |
|  | Cuban dry forests | 65614.90 | 28 | 1.19 |  | X |  | |
|  | Ecuadorian dry forests | 21188.70 | 118 | 3.92 |  | X |  | |
|  | Hispaniolan dry forests | 15446.76 | 16 | 0.64 |  |  | X | |
|  | Islas Revillagigedo dry forests | 213.41 | 1 | 0.00 | X |  |  | |
|  | Jalisco dry forests | 26051.22 | 128 | 5.32 | X |  |  | |
|  | Jamaican dry forests | 2309.70 | 18 | 0.90 |  |  | X | |
|  | Lara-Falcón dry forests | 16871.01 | 152 | 5.16 |  | X |  | |
|  | Leeward Islands dry forests | 148.29 | 11 | 0.71 |  | X |  | |
|  | Magdalena Valley dry forests | 19550.17 | 192 | 7.14 |  | X |  | |
|  | Maracaibo dry forests | 30099.99 | 176 | 6.56 |  | X |  | |
|  | Marañón dry forests | 11323.06 | 161 | 6.14 | X |  |  | |
|  | Panamanian dry forests | 5087.84 | 142 | 4.14 |  |  | X | |
|  | Patia Valley dry forests | 2260.75 | 156 | 5.62 |  | X |  | |
|  | Puerto Rican dry forests | 1271.24 | 11 | 0.64 |  |  | X | |
|  | Sierra de la Laguna dry forests | 3975.18 | 39 | 1.74 | X |  |  | |
|  | Sinaloan dry forests | 77364.29 | 150 | 6.26 | X |  |  | |
|  | Sin· Valley dry forests | 24880.78 | 166 | 6.45 |  |  | X | |
|  | Southern Pacific dry forests | 42283.80 | 167 | 5.42 |  | X |  | |
|  | Trinidad and Tobago dry forests | 270.41 | 79 | 2.56 |  | X |  | |
|  | Tumbes-Piura dry forests | 41103.15 | 120 | 5.72 | X |  |  | |
|  | Veracruz dry forests | 6616.06 | 108 | 3.37 |  | X |  | |
|  | Windward Islands dry forests | 490.12 | 23 | 0.65 |  | X |  | |
|  | Yucatán dry forests | 49625.62 | 70 | 3.1 |  | X |  | |
| T&S coniferous forests | Bahamian pine forests | 2085.63 | 7 | 0.60 |  |  | X | |
|  | Belizian pine forests | 2822.09 | 101 | 3.30 |  | X |  | |
|  | Central American pine-oak forests | 110948.69 | 195 | 4.82 |  | X |  | |
|  | Cuban pine forests | 6404.99 | 26 | 1.19 |  | X |  | |
|  | Hispaniolan pine forests | 11560.74 | 16 | 0.64 |  |  | X | |
|  | Miskito pine forests | 18845.14 | 101 | 3.09 |  | X |  | |
|  | Sierra Madre de Oaxaca pine-oak forests | 14299.06 | 171 | 5.50 |  | X |  | |
|  | Sierra Madre del Sur pine-oak forests | 60976.86 | 150 | 4.86 |  | X |  | |
|  | Sierra de la Laguna pine-oak forests | 1061.10 | 39 | 1.74 | X |  |  | |
|  | Trans-Mexican Volcanic Belt pine-oak forests | 91553.94 | 191 | 7.69 | X |  |  | |
| Temp. broadleaf and mixed forests | Juan Fernandez Islands temperate forests | 145.85 | 2 | 0.50 | X |  |  | |
|  | Magellanic subpolar forests | 147802.60 | 33 | 0.81 | X |  |  | |
|  | San Felix-San Ambrosio Islands temperate forests | 6.49 | 2 | 0.50 | X |  |  | |
|  | Valdivian temperate forests | 248398.14 | 55 | 1.47 | X |  |  | |
| T&S grasslands, savannas, and shrublands | Arid Chaco | 98764.40 | 61 | 2.12 | X |  |  | |
|  | Beni savanna | 125596.96 | 192 | 7.52 |  | X |  | |
|  | Campos Rupestres montane savanna | 26314.13 | 182 | 5.38 | X |  |  | |
|  | Cerrado | 1910153.42 | 278 | 8.27 | X |  |  | |
|  | Córdoba montane savanna | 58097.67 | 70 | 2.51 | X |  |  | |
|  | Guyanan savanna | 103896.12 | 207 | 7.89 |  |  | X | |
|  | Humid Chaco | 334130.27 | 135 | 4.37 | X |  |  | |
|  | Llanos | 387336.59 | 253 | 9.41 |  | X |  | |
|  | Uruguayan savanna | 355387.46 | 106 | 3.45 | X |  |  | |
|  | clipperton Island Scrub and Grassland | 3.92 | 0 | 0 | X |  |  | |
| Temp. grasslands, savannas, and shrublands | Argentine Espinal | 108723.72 | 57 | 3.31 | X |  |  | |
|  | Argentine Monte | 409134.31 | 99 | 3.27 | X |  |  | |
|  | Humid Pampas | 240796.09 | 60 | 2.20 | X |  |  | |
|  | Patagonian grasslands | 63412.91 | 28 | 0.71 | X |  |  | |
|  | Patagonian steppe | 488386.15 | 50 | 1.33 | X |  |  | |
|  | Semi-arid Pampas | 327113.02 | 58 | 1.87 | X |  |  | |
| Flooded grasslands and savannas | Central Mexican wetlands | 278.32 | 98 | 4.24 | X |  |  | |
|  | Cuban wetlands | 5649.27 | 26 | 1.19 |  | X |  | |
|  | Enriquillo wetlands | 628.49 | 15 | 0.64 |  |  | X | |
|  | Everglades | 20029.02 | 39 | 1.42 |  | X |  | |
|  | Guayaquil flooded grasslands | 2924.09 | 103 | 4.66 | X |  |  | |
|  | Orinoco wetlands | 5988.95 | 162 | 5.53 |  | X |  | |
|  | Pantanal | 170511.50 | 162 | 6.52 | X |  |  | |
|  | Paraná flooded savanna | 38840.08 | 72 | 3.29 | X |  |  | |
|  | Southern Cone Mesopotamian savanna | 77546.19 | 95 | 3.08 | X |  |  | |
| Montane grasslands and shrublands | Central Andean dry puna | 306574.78 | 113 | 4.97 | X |  |  | |
|  | Central Andean puna | 160930.16 | 196 | 5.94 |  | X |  | |
|  | Central Andean wet puna | 116881.45 | 223 | 6.78 |  | X |  | |
|  | Cordillera Central paramo | 12121.34 | 167 | 6.57 | X |  |  | |
|  | Cordillera de Merida paramo | 2797.80 | 175 | 6.45 |  | X |  | |
|  | Northern Andean paramo | 29838.34 | 281 | 10.25 |  | X |  | |
|  | Santa Marta paramo | 1238.77 | 145 | 4.98 |  |  | X | |
|  | Southern Andean steppe | 178094.71 | 69 | 1.95 | X |  |  | |
|  | Zacatonal | 301.25 | 153 | 6.33 | X |  |  | |
| Mediterranean forests, woodlands, and scrub or sclerophyll forests | Chilean matorral | 148382.78 | 40 | 1.16 | X |  |  | |
| Deserts and xeric shrublands | Araya and Paria xeric scrub | 5260.67 | 154 | 5.45 |  | X |  | |
|  | Aruba-Curacao-Bonaire cactus scrub | 456.77 | 8 | 0.81 |  | X |  | |
|  | Atacama desert | 104908.28 | 32 | 1.12 | X |  |  | |
|  | Caatinga | 731370.19 | 170 | 6.44 | X |  |  | |
|  | Cuban cactus scrub | 3255.85 | 26 | 1.19 |  |  | X | |
|  | Galapagos Islands xeric scrub | 7978.88 | 6 | 0.00 |  |  | X | |
|  | Guajira-Barranquilla xeric scrub | 31479.67 | 172 | 6.60 |  |  | X | |
|  | La Costa xeric shrublands | 68185.81 | 181 | 6.50 |  | X |  | |
|  | Leeward Islands xeric scrub | 1637.64 | 14 | 0.79 |  | X |  | |
|  | Malpelo Island xeric scrub | 7.66 | 1 | 0.00 |  |  | X | |
|  | Motagua Valley thornscrub | 2328.50 | 128 | 3.93 |  | X |  | |
|  | Paraguana xeric scrub | 15910.13 | 165 | 5.96 |  | X |  | |
|  | San Lucan xeric scrub | 3867.40 | 42 | 1.84 | X |  |  | |
|  | Sechura desert | 184225.44 | 125 | 4.76 |  | X |  | |
|  | Tehuacan Valley matorral | 9862.23 | 133 | 4.35 |  | X |  | |
|  | Windward Islands xeric scrub | 1022.79 | 26 | 0.97 |  | X |  | |
| Mangrove | Alvarado mangroves | 4534.86 | 135 | 4.32 |  | X |  | |
|  | Amapa mangroves | 1555.28 | 146 | 5.39 |  |  | X | |
|  | Bahamian mangroves | 6605.16 | 10 | 0.56 |  |  | X | |
|  | Bahia mangroves | 2112.10 | 135 | 3.93 | X |  |  | |
|  | Belizean Coast mangroves | 2785.04 | 113 | 3.59 |  | X |  | |
|  | Belizean Reef mangroves | 236.24 | 0 | 0.00 |  | X |  | |
|  | Bocas del Toro-San Bastimentos Island-San Blas mangroves | 538.77 | 143 | 4.06 |  |  | X | |
|  | Coastal Venezuelan mangroves | 5819.85 | 181 | 6.41 |  | X |  | |
|  | Esmeraldes/Chocó mangroves | 6488.74 | 146 | 3.86 |  | X |  | |
|  | Greater Antilles mangroves | 10618.21 | 38 | 2.11 |  |  | X | |
|  | Guianan mangroves | 14507.80 | 204 | 7.45 |  |  | X | |
|  | Gulf of Fonseca mangroves | 1618.83 | 103 | 5.34 | X |  |  | |
|  | Gulf of Guayaquil-Tumbes mangroves | 3298.94 | 96 | 3.80 | X |  |  | |
|  | Gulf of Panama mangroves | 2413.89 | 168 | 4.75 |  |  | X | |
|  | Ilha Grande mangroves | 3200.25 | 145 | 4.23 | X |  |  | |
|  | Lesser Antilles mangroves | 649.50 | 21 | 0.81 |  | X |  | |
|  | Magdalena-Santa Marta mangroves | 3181.27 | 170 | 5.15 |  |  | X | |
|  | Manabi mangroves | 1143.67 | 89 | 3.24 |  | X |  | |
|  | Maranhao mangroves | 11259.07 | 137 | 6.15 | X |  |  | |
|  | Marismas Nacionales-San Blas mangroves | 2034.76 | 87 | 3.74 | X |  |  | |
|  | Mayan Corridor mangroves | 4079.71 | 86 | 3.06 |  | X |  | |
|  | Mexican South Pacific Coast mangroves | 1168.25 | 118 | 3.52 | X |  |  | |
|  | Moist Pacific Coast mangroves | 1591.01 | 133 | 3.31 |  |  | X | |
|  | Mosquita-Nicaraguan Caribbean Coast mangroves | 4421.05 | 107 | 3.21 |  | X |  | |
|  | Northern Dry Pacific Coast mangroves | 1053.11 | 94 | 5.15 |  | X |  | |
|  | Northern Honduras mangroves | 1051.69 | 114 | 2.92 |  | X |  | |
|  | Para mangroves | 4393.68 | 142 | 5.06 |  |  | X | |
|  | Petenes mangroves | 1971.66 | 62 | 2.65 |  | X |  | |
|  | Piura mangroves | 116.74 | 45 | 1.51 | X |  |  | |
|  | Rio Lagartos mangroves | 3457.18 | 64 | 2.75 |  | X |  | |
|  | Rio Negro-Rio San Sun mangroves | 475.19 | 122 | 2.88 |  |  | X | |
|  | Rio Piranhas mangroves | 2105.76 | 121 | 5.90 | X |  |  | |
|  | Rio Sao Francisco mangroves | 2607.43 | 129 | 6.29 | X |  |  | |
|  | Southern Dry Pacific Coast mangroves | 902.23 | 125 | 3.09 | X |  |  | |
|  | Tehuantepec-El Manchon mangroves | 2685.10 | 120 | 4.94 | X |  |  | |
|  | Trinidad mangroves | 185.01 | 78 | 2.46 |  | X |  | |
|  | Usumacinta mangroves | 3118.62 | 79 | 2.67 |  | X |  | |
| Total |  |  |  |  | 82 | 88 | 49 | |
